# Validating Fitbit Zip for monitoring physical activity of children in school: a cross-sectional study

**DOI:** 10.1186/s12889-018-5752-7

**Published:** 2018-07-11

**Authors:** Kerli Mooses, Marek Oja, Sulev Reisberg, Jaak Vilo, Merike Kull

**Affiliations:** 1 Institute of Sport Sciences and Physiotherapy, University of Tartu, Jakobi 5, Tartu, Estonia; 2 Software Technology and Applications Competence Centre, Ülikooli 2, Tartu, Estonia; 3 Institute of Computer Science, University of Tartu, J. Liivi 2, Tartu, Estonia; 4 Quretec Ltd, Ülikooli 6a, Tartu, Estonia

**Keywords:** Activity monitor, Fitbit zip, Accelerometer, Validity

## Abstract

**Background:**

Modern activity trackers, including the Fitbit Zip, enable the measurement of both the step count as well as physical activity (PA) intensities. However, there is a need for field-based validation studies in a variety of populations before using trackers for research. Therefore, the purpose of the current study was to investigate the validity of Fitbit Zip step count, moderate to vigorous physical activity (MVPA) and sedentary minutes, in different school segments in 3rd grade students.

**Methods:**

Third grade students (*N* = 147, aged 9–10 years) wore a Fitbit Zip and an ActiGraph GT3x-BT accelerometer simultaneously on a belt for five days during school hours. The number of steps, minutes of MVPA and sedentary time during class time, physical education lessons and recess were extracted from both devices using time filters, based on the information from school time tables obtained from class teachers. The validity of the Fitbit Zip in different school segments was assessed using Bland-Altman analysis and Spearman’s correlation.

**Results:**

There was a strong correlation in the number of steps in all in-school segments between the two devices (*r* = 0.85–0.96, *P* < 0.001). The Fitbit Zip overestimated the number of steps in all segments, with the greatest overestimation being present in physical education lessons (345 steps). As for PA intensities, the agreement between the two devices in physical education and recess was moderate for MVPA minutes (*r* = 0.56 and *r* = 0.72, *P* < 0.001, respectively) and strong for sedentary time (*r* = 0.85 and *r* = 0.87, *P* < 0.001, respectively). During class time, the correlation was weak for MVPA minutes (*r* = 0.24, *P* < 0.001) and moderate for sedentary time (*r* = 0.57, *P* < 0.001). For total in-school time, the correlation between the two devices was strong for steps (*r* = 0.98, *P* < 0.001), MVPA (*r* = 0.80, *P* < 0.001) and sedentary time (*r* = 0.94, *P* < 0.001).

**Conclusion:**

In general, the Fitbit Zip can be considered a relatively accurate device for measuring the number of steps, MVPA and sedentary time in students in a school-setting. However, in segments where sedentary time dominates (e.g. academic classes), a research-grade accelerometer should be preferred.

## Background

Despite the abundance of evidence showing the positive influence of physical activity (PA) on physical, social and mental health [1, 2], the physical activity levels of children remain low [3, 4]. In order to assess adherence to PA guidelines and provide accurate measures concerning the amount of PA, valid, reliable and feasible measurement instruments are needed.

In general, pedometers are accepted as a valid tool for objectively measuring the PA of children [5]. Therefore, they are widely used in measuring both overall PA as well as PA in different settings [6–9]. Due to their relatively low cost, attractiveness, and ease of use, consumer-grade pedometers are an appealing alternative to research-grade accelerometers. One shortcoming of many pedometers is their limited ability to count only the number of steps but not the intensity of the exercise. Therefore, it is difficult to assess compliance with PA guidelines, which are based on the intensity and duration of PA, recommending a minimum of 60 min of moderate to vigorous physical activity (MVPA) every day [10]. There have been attempts to find step counts that correspond to the recommended 60 min of MVPA. As a result, a desired number of daily steps has been proposed to range from 10,000 to 15,000 steps/day [11, 12]. As students spend a lot of time at school, it has been advised that half of the 60 min daily recommendation should be acquired in school [13]. It has been indicated that 5300 steps for girls and 5800 steps for boys can determine whether the students have achieved the recommended 30 min of MVPA in school [14].

More recently, consumer-grade monitors that measure the intensity of PA in addition to simple step count are rapidly appearing. Therefore, they have the potential to be used in studies evaluating students’ adherence with PA recommendations as well as the amount of MVPA minutes acquired in different school day segments (i.e. class time, recess, physical education lesson). One such consumer monitor, frequently used in a research setting, is the Fitbit Zip, as it has shown to be valid in terms of step count both in laboratory [15, 16] and free-living conditions in adults [15–18]. As for PA intensities, a study with adults has indicated a strong correlation in MVPA minutes between the Fitbit Zip and the ActiGraph [17]. It is also important that subjects rate the Fitbit Zip acceptable to use and easy to integrate into their daily routine [18], therefore increasing the feasibility of their use in larger studies. Still, the need for more field-based studies in a variety of populations has been stressed in a recent review [16]. Moreover, there is little published data on validation studies conducted among children and youth. To the best of our knowledge there is only one study validating the step count of the Fitbit Zip among free-living adolescents, and thus confirming the previous studies with adult population and showing the Fitbit Zip to be a valid tool for measuring step count [19]. The reliability and validity of Fitbit Zip trackers has also been confirmed in preschool (3–4 year-olds) children in a childcare setting [20]. Still, there is a lack of research validating Fitbit Zip steps counts or PA intensities in school children. Therefore, before using this device in research, the validation of Fitbit Zip estimates in comparison with accelerometer estimates is needed.

Therefore, the main aim of the current study was to investigate the validity of Fitbit Zip step count, MVPA and sedentary minutes in different school segments in children.

## Methods

### Study design

A cross-sectional study was conducted to assess the validity of Fitbit Zip in measuring step count, MVPA and sedentary time in school setting, compared to a previously validated research-level accelerometer.

### Devices

The Fitbit Zip (Fitbit Inc., USA) is a small, light (8 g) and relatively inexpensive belt-worn activity monitor (Table 1). Data from the device can be transferred to a computer via Bluetooth and its replaceable battery runs for up to 6 months, making it a convenient device for measuring physical activity for both users and researchers. The Fitbit Zip has been previously shown to be a valid instrument for counting steps in free-living setting in adult, adolescent, and preschool populations [15–20].

**Table 1:** Description of Fitbi Zip and ActiGraph GT3x-BT

	Fitbit Zip	ActiGraph GT3x
Internal sensor	3 axis accelerometer	3 axis accelerometer + ambient light sensor
Output	Number of steps Minutes of fairly active, very active, light, sedentary	Number of steps Minutes of vigorous, moderate, light, sedentary
Communication	Bluetooth	USB, Bluetooth
Memory size	7 days of detailed motion data	4 GB
Battery	Replaceable	Rechargeable
Battery life	Up to 6 months	25 days
Size	2.8 × 1.0 × 3.6 cm	4.6 × 3.3 × 1.5 cm
Weight	8 g	19 g
Attachment site	Hip	Hip, wrist, ankle, thigh
Display screen	Yes	No
Cost	Approx. 60 EUR	Approx. 250 EUR

The ActiGraph GT3x-BT (ActiGraph LLC, Pensacola, FL, USA) is a research grade tri-axial accelerometer that can be attached on the belt with a rubber band. The device is rechargeable, and the initialization and data download to computer is performed via USB. We used the ActiGraph GT3x-BT accelerometer to validate the output of the Fitbit Zip as ActiGraph GT3x-BT has been shown to be a valid device for measuring the physical activity of children [21] and it has been previously widely used in physical activity research with children [22–24].

### Participants

The sample consisted of third grade students (aged 9–10 years) from seven Estonian schools participating in a pilot project that aimed at increasing the physical activity of students in the school setting. The pilot schools were situated in different parts of Estonia and varied according to their ownership (private vs municipality schools), location (city vs countryside), and size (from 175 to 789 students).

### Procedure

The study was performed in accordance with the Declaration of Helsinki and approved by the Research Ethics Committee of the University of Tartu (nr 255/M-11). Written informed consent was received from all schools, parents and students willing to participate (*N* = 219) resulting in a participation rate of 92%. From all consented students a subsample (*N* = 147) was formed for measuring physical activity with the Fitbit Zip and ActiGraph simultaneously.

The accelerometer and Fitbit Zip were attached on the hip with the same elastic belt and worn on the same side. The accelerometer was set to record physical activity data in 15 s intervals. Students wore the devices for one school week in September and another school week in November 2016. The devices were worn only during school hours – they were distributed to the students before the beginning of first lesson and collected after the last lesson by class teachers. The devices were stored in a plastic bag with a special sticker on it to enable the students to recognize their devices easily every day.

School timetables and information concerning school attendance were obtained from class teachers in order to match the PA to the classes of specific school subjects.

### Statistical analysis

Physical activity data from accelerometers were downloaded and processed using ActiLife software version 6.11.2 (ActiGraph LLC, Pensacola, FL, USA). To calculate minutes spent in sedentary (≤100 counts per minute), light (101–2295 counts per minute) and MVPA (≥2296 counts per minute), Evenson cut-points were used [25], as they have shown the best classification accuracy in children [25]. Zero counts for a consecutive 60 min were classified as non-wear time.

Data from Fitbit Zip devices were first automatically synced to a Fitbit server and then downloaded to the SQL-based Qure Data Management Platform via the Fitbit application programming interface (API). As Fitbit data was collected in 1 min intervals, a Python script was used to aggregate the data to longer intervals having the length of one lesson or recess. “Fairly active” and “very active” intensities from Fitbit were considered as moderate and vigorous activity respectively.

The number of steps, minutes of MVPA and sedentary time during lessons and recess were extracted using time filters, based on the information from school time tables. As a result of data preparation, the data set contained the following information: subject ID, gender, school ID, class ID, date, day of the week, type of interval (class time, physical education lesson, recess), length of interval in minutes, step counts, sedentary and active (light, moderate and vigorous) minutes for Fitbit and ActiGraph devices. Data was also checked for outliers. In total, there were 8923 rows of data. First, 923 rows (10.3%) were removed from data due to Fitbit time inaccuracies (shifts in Fitbit device clocks due to unknown reasons so that we were unable to compare the data to ActiGraph within the same lessons). In addition, 515 data rows (5.8%) were removed because their step count by Fitbit was zero. For all of those 515 data rows, the Fitbit step count was zero for whole day, indicating a faulty measurement. Finally, after careful examination of the raw data and in order to remove extreme outliers, we left out 32 rows (0.4%) from the data, where the difference between the steps by Fitbit and ActiGraph differed more than 10 times. As a result, the cleaned dataset contained 7453 observations – 3850 for class time, 309 for physical education lessons and 3294 for recess – of 144 students.

Descriptive statistics were used to characterize the sample and physical activity during class time, physical education lessons and recess. The data was checked for normality using the Shapiro-Wilks test. To assess the potential systematic difference between two devices the Wilcoxon Signed Rank test was used. Spearman’s correlation coefficient r was calculated in order to compare the number of steps, minutes of MVPA and sedentary time derived by the Fitbit Zip and ActiGraph. To test the agreement between two devices, Bland-Altman analysis was applied, where in addition to the limits of agreement, the bias between two devices was calculated. The bias is the mean of differences of the two devices. Positive bias indicates that the Fitbit Zip systematically overestimates the number of steps or activity minutes compared to the ActiGraph accelerometer. The data analysis was conducted by using R version 3.2.3.

## Results

The gender distribution in the final sample was equal (72 males and 72 females). The physical activity of students in class, physical education lessons and recess is summarised in Table 2. The most active segment during school day was the physical education lesson, where on average more than 2000 steps and 15 min of MVPA were acquired. The class time remained mostly sedentary – over 33 min (out of a 45 min lesson) was spent as sedentary on average.

**Table 2:** Mean (SD), correlations and Bland-Altman output for Fitbit Zip and ActiGraph in school segments

	Fitbit Zip	ActiGraph GT3x	*r*	Bias	LoA	LuA
Steps						
Class time	231.5 (298.2)	206.2 (222.9)	0.85**	25.2	− 209.6	260.3
Physical education lesson	2354.0 (1279.6)	2008.7 (1172.5)**	0.96**	345.3	− 163.8	854.4
Recess	472.2 (337.2)	388.5 (268.2)**	0.96**	83.7	−127.3	294.7
MVPA (min)						
Class time	0.6 (2.5)	0.9 (1.7)**	0.24**	−0.3	−4.0	3.3
Physical education lesson	17.8 (14.1)	15.4 (9.1)*	0.72**	2.4	−14.7	19.4
Recess	2.1 (4.3)	2.4 (2.4)**	0.56**	−0.3	−6.4	5.8
Sedentary time (min)						
Class time	37.5 (5.8)	33.8 (6.5)**	0.57**	3.6	−6.5	13.8
Physical education lesson	11.1 (9.8)	13.7 (9.0)**	0.85**	−2.6	−10.4	5.2
Recess	5.5 (4.1)	5.4 (3.9)	0.87**	0.1	−3.7	3.9

Note: * *P* < 0.05, ** *P* < 0.001, *LoA* lower limits of agreement, *LuA* upper limits of agreement. Differences between two devices according to Wilcoxon Signed Rank test are presented in column “ActiGraph GT3x”

When comparing the two devices, there was a strong correlation in the number of steps in all in-school segments between the Fitbit Zip and ActiGraph (*r* = 0.85–0.96, *P* < 0.001) (Table 2). The number of steps between the two devices were significantly different during physical education lessons and recess. Bland-Altman analyses indicated that the Fitbit Zip systematically overestimated the number of steps compared to ActiGraph accelerometer, with the greatest difference occurring during physical education lessons.

The minutes of MVPA in different in-school segments differed between two devices, while the correlation was moderate during physical education lesson and recess (*r* = 0.56–0.72, *P* < 0.001) and weak (*r* = 0.24*, P* < 0.001) during class time. The Fitbit Zip underestimated the amount of MVPA during class time and recess, and overestimated it during physical education lessons.

As for sedentary time, there was a moderate to strong correlation (*r* = 0.57–0.87, *P* < 0.001) between two devices depending on the segment. The Fitbit overestimated time spent as sedentary in classes and during in-school segment compared to the ActiGraph, while in physical education lessons, the Fitbit underestimated sedentary time.

When looking at total in-school activity, students accumulated more than 21.7–25.6% of daily recommended physical activity in school (Table 3). During school hours, students spent more than 1.5 h sedentary.

**Table 3:** Physical activity during school hours (means (SD)) and proportion from daily physical activity recommendation

	Fitbit Zip	ActiGraph GT3x
Steps	3069.6 (1978.8)	2605.8 (1652.1)*
% from recommendations^a^	25.6	21.7*
MVPA (min)	14.3 (17.0)	15.8 (12.9)*
% from recommendations^b^	23.8	26.3*
Sedentary time (min)	160.4 (59.3)	147.4 (56.9)*

Note: *P < 0.001; a – recommendation: 12000 steps/day; b – recommendation: 60 min of moderate

**Fig. 1:**
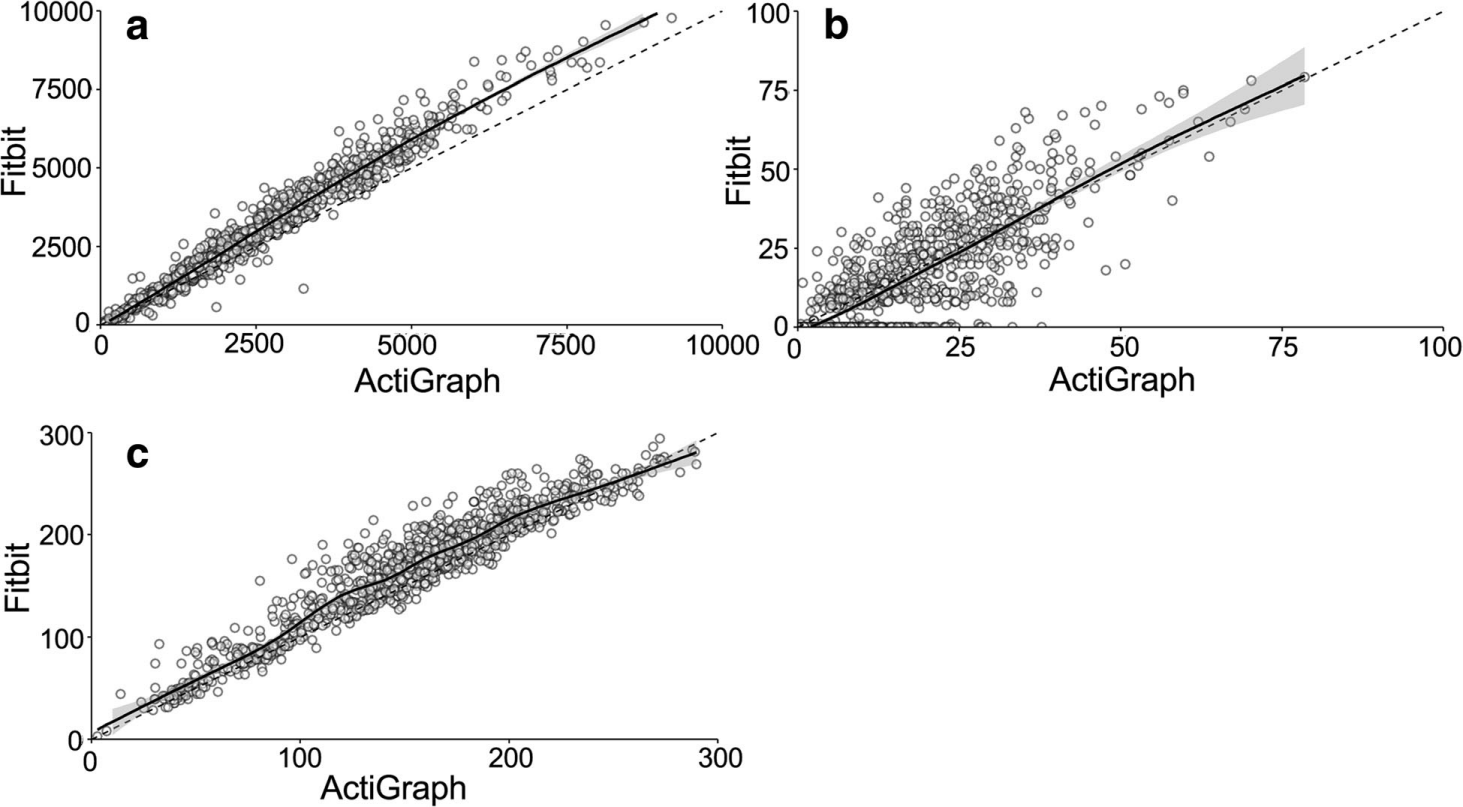
Correlations between the steps **a**, minutes of MVPA (**b**) and sedentary time (**c**) of ActiGraph and Fitbit. Solid line is the loess curve based on the data and dashed line represents situation where *r* = 1

For total in-school time, the correlation between two devices was strong for steps (*r* = 0.98, *P* < 0.001), MVPA (*r* = 0.80, *P* < 0.001) and sedentary time (*r* = 0.94, *P* < 0.001) (Fig. 1). According to Bland-Altman analyses the Fitbit Zip overestimated both the number of steps (bias: 464 (− 446–1374) steps) and sedentary time (bias: 13.1 (− 21.0–47.1) minutes) and underestimated MVPA minutes (bias: − 1.5 (− 20.2–17.3) minutes) compared to the ActiGraph accelerometer.

## Discussion

Consumer-level monitors make the devices more accessible to researchers, however there is a need for validation studies, especially among children. Therefore, the aim of this study was to fill this gap in scientific literature and validate the step count, MVPA and sedentary time of Fitbit Zip among third grade students (9–10 year-old) in a school setting.

In general, the Fitbit Zip can be considered a relatively accurate device for measuring steps in third grade students in school, despite the fact that the Fitbit Zip tended to overestimate the number of steps during school hours as well as during all in-school segments (class time, physical education lessons, and recess). However, the amount of overestimation was small. The overestimation of steps by the Fitbit Zip is in line with previous studies where daily physical activity of adults [17, 18], adolescents [19] or preschool children [20] has been studied. Despite the overestimation, the correlation between the Fitbit Zip and ActiGraph was strong in all in-school segments, supporting previous research [16–19]. Therefore, the findings of the present study extended previous research and confirmed that the Fitbit Zip is a valid device for measuring steps also in students.

In addition to the number of steps, several modern activity monitors, including the Fitbit Zip, record information about physical activity intensity that can be used to determine the accumulation of recommended minutes of MVPA. However, research on the validity of intensities measured with the Fitbit Zip is especially lacking in children. Therefore, the unique aspect of the current study is the comparison of minutes of MVPA and sedentary time between the Fitbit Zip and a research-grade accelerometer. When looking at total in-school time, it can be concluded that the Fitbit Zip can be used for measuring both sedentary time and MVPA in school, as the bias between two devices was small and the correlation with ActiGraph was strong. These results concerning the MVPA minutes support previous research with adults [17].

The validity of Fitbit Zip activity minutes can depend on in-school segments. One unexpected finding was that the Fitbit Zip performed weaker in the classroom setting, where sedentary behaviour dominated. During classes, the Fitbit Zip underestimated the minutes of MVPA, overestimated sedentary time, and was weakly to moderately correlated with ActiGraph values. It could be hypothesised that one reason for the modest agreement between two devices was due to the sedentary nature of academic classes combined with the differences in data recording intervals (15-s for ActiGraph and 1-min for Fitbit Zip), which makes the ActiGraph more sensitive to short activity bouts. In classes, the sedentary behaviour dominated when over 33 min out of 45-min lesson was spent as sedentary and less than one MVPA minute was acquired. As for physical education lessons and recess, both of which are more physically active segments, the validity of Fitbit Zip was acceptable, as the bias between Fitbit Zip and ActiGraph was smaller. Moreover, the minutes of MVPA and sedentary time were moderately to strongly correlated.

It has been proposed that due to their relatively low cost and usability, activity trackers could be used in scientific research [15, 18, 19]. In general, the current study supports this position. It has been suggested that strong correlations provide preliminary evidence of the reasonable validity of consumer-level devices [17]. Therefore, our study indicated that the Fitbit Zip is suitable for measuring in-school steps as well as MVPA minutes of students. Moreover, the Fitbit Zip can be considered an acceptable, enabling to determine the adherence to PA recommendations as well as the contribution of school day PA to daily PA. However, caution should be applied when the aim is to measure day segments in which sedentary behaviour dominates (e.g. class settings). In such case more sensitive research-grade devices should be favoured. In addition, it has been suggested that activity trackers might not be suitable for longer interventions as the manufacturer does not inform the users in case of a change in its algorithms [19]. Another limitation for researchers is the data processing options offered by Fitbit Inc. Currently, only daily aggregated data can be downloaded via Fitbit online software, while in research, more detailed data is often preferred and, moreover, more detailed data is recorded and uploaded to Fitbit’s server. Therefore, researchers have to make additional efforts to find ways to download the detailed data (e.g. develop application for using Fitbit API). It should also be noted that the Fitbit Zip does not detect non-wear time. Thus, in case of daily data measurements, the activity trackers should be accompanied with a diary in order to minimize possible bias in sedentary time evaluation.

One limitation of the study is the absence of after-school data which could have enabled to assess compliance with daily PA recommendations. However, by focusing only on in-school time by attaching trackers to students only when they were at school, we reduced the risk of losing or forgetting devices and therefore increased the data quality. Still, we lost 1470 data rows (16.4% of the initial database) due to device malfunction, which is similar to a review reporting a 11–33% of data loss for field-based studies with activity trackers [16]. The strengths of our study are the free-living setting and assessing PA intensities in addition to step counts. This represents a significant contribution over previous research, as there is a lack of studies examining the validity of MVPA in school children from different age groups, and to the best of our knowledge, no study has examined the validity of sedentary time of Fitbit Zip in comparison to ActiGraph in school children. As sometimes cheaper devices cannot be avoided when conducting a study, future research should also incorporate a wider range of consumer-grade activity trackers from different manufacturers in order to support the evidence-based decision of researchers when choosing the device.

## Conclusion

In summary, the evidence from this study shows that the Fitbit Zip can be considered a valid device for measuring the steps, MVPA and sedentary time of students in a school setting. However, in segments where sedentary time dominates (e.g. academic lessons), a research-grade accelerometer should be preferred. Present study is a valuable addition to previous validation studies conducted with adults, adolescents, and preschool children, confirming that the Fitbit Zip can be used as an alternative to a research-grade accelerometer for measuring both the steps as well as the physical activity intensities in 9–10 year-old children.

## Abbreviations

MVPA: Moderate to vigorous physical activity
PA: Physical activity

## References

[1] Janssen I, Leblanc AG (2010). Systematic review of the health benefits of physical activity and fitness in school-aged children and youth. Int J Behav Nutr Phys Act.

[2] Poitras VJ, Gray CE, Borghese MM, Carson V, Chaput J-P, Janssen I (2016). Systematic review of the relationships between objectively measured physical activity and health indicators in school-aged children and youth. Appl Physiol Nutr Metab.

[3] Tremblay MS, Barnes JD, Gonzalez SA, Katzmarzyk PT, Onywera VO, Reilly JJ (2016). Global matrix 2.0: report card grades on the physical activity of children and youth comparing 38 countries. J Phys Act Health.

[4] Roman-Viñas B, Chaput J-P, Katzmarzyk PT, Fogelholm M, Lambert EV, Maher C (2016). Proportion of children meeting recommendations for 24-hour movement guidelines and associations with adiposity in a 12-country study. Int J Behav Nutr Phys Act.

[5] McNamara E, Hudson Z, Taylor SJC (2010). Measuring activity levels of young people: the validity of pedometers. Br Med Bull.

[6] Duncan MJ, Al-Nakeeb Y, Woodfield L, Lyons M (2007). Pedometer determined physical activity levels in primary school children from Central England. Prev Med.

[7] Cox M, Schofield G, Greasley N, Kolt GS (2006). Pedometer steps in primary school-aged children: a comparison of school-based and out-of-school activity. J Sci Med Sport.

[8] Tudor-Locke C, McClain JJ, Hart TL, Sisson SB, Washington TL (2009). Expected values for pedometer-determined physical activity in youth. Res Q Exerc Sport.

[9] Tudor-Locke C, Lee SM, Morgan CF, Beighle A, Pangrazi RP (2006). Children’s pedometer-determined physical activity during the segmented school day. Med Sci Sports Exerc.

[10] WHO (2015). Physical activity strategy for the WHO European Region 2016–2025.

[11] Tudor-Locke C, Craig CL, Beets MW, Belton S, Cardon GM, Duncan S (2011). How many steps/day are enough? For children and adolescents. Int J Behav Nutr Phys Act.

[12] Colley RC, Janssen I, Tremblay MS (2012). Daily step target to measure adherence to physical activity guidelines in children. Med Sci Sports Exerc.

[13] Pate RR, Davis MG, Robinson TN, Stone EJ, McKenzie TL, Young JC (2006). Promoting physical activity in children and youth: a leadership role for schools: a scientific statement from the American Heart Association Council on nutrition, physical activity, and metabolism (physical activity committee) in collaboration with the councils on cardiovascular disease in the young and cardiovascular nursing. Circulation.

[14] Burns RD, Brusseau TA, Fu Y, Hannon JC (2016). Establishing school day pedometer step count cut-points using ROC curves in low-income children. Prev Med.

[15] Kooiman TJM, Dontje ML, Sprenger SR, Krijnen WP, van der Schans CP, de Groot M (2015). Reliability and validity of ten consumer activity trackers. BMC Sports Sci Med Rehabil.

[16] Evenson KR, Goto MM, Furberg RD (2015). Systematic review of the validity and reliability of consumer-wearable activity trackers. Int J Behav Nutr Phys Act.

[17] Ferguson T, Rowlands AV, Olds T, Maher C (2015). The validity of consumer-level, activity monitors in healthy adults worn in free-living conditions: a cross-sectional study. Int J Behav Nutr Phys Act.

[18] Tully MA, McBride C, Heron L, Hunter RF (2014). The validation of Fitbit zip™ physical activity monitor as a measure of free-living physical activity. BMC Res Notes..

[19] Schneider M, Chau L (2016). Validation of the Fitbit zip for monitoring physical activity among free-living adolescents. BMC Res Notes.

[20] Sharp CA, Mackintosh KA, Erjavec M, Pascoe DM, Horne PJ (2017). Validity and reliability of the Fitbit zip as a measure of preschool children’s step count. BMJ Open Sport Exerc Med.

[21] Trost SG, Loprinzi PD, Moore R, Pfeiffer KA (2011). Comparison of accelerometer cut points for predicting activity intensity in youth. Med Sci Sports Exerc.

[22] Guinhouya BC, Samouda H, de Beaufort C (2013). Level of physical activity among children and adolescents in Europe: a review of physical activity assessed objectively by accelerometry. Public Health.

[23] Mooses K, Mägi K, Riso E-M, Kalma M, Kaasik P, Kull M (2017). Objectively measured sedentary behaviour and moderate and vigorous physical activity in different school subjects: a cross-sectional study. BMC Public Health.

[24] Konstabel K, Veidebaum T, Verbestel V, Moreno LA, Bammann K, Tornaitis M (2014). Objectively measured physical activity in European children: the IDEFICS study. Int J Obes.

[25] Evenson KR, Catellier DJ, Gill K, Ondrak KS, McMurray RG (2008). Calibration of two objective measures of physical activity for children. J Sports Sci.

